# Relationship between spinal structural damage on radiography and bone fragility on CT in ankylosing spondylitis patients

**DOI:** 10.1038/s41598-021-88838-9

**Published:** 2021-04-29

**Authors:** Marine Fauny, Frank Verhoeven, Edem Allado, Eliane Albuisson, Astrid Pinzano, Caroline Morizot, Isabelle Chary-Valckenaere, Damien Loeuille

**Affiliations:** 1 Department of Rheumatology, Hôpitaux de Brabois, Nancy University Hospital, 54511 Vandoeuvre les Nancy Cedex, France; 2 Saint Charles Hospital, 54200 Toul, France; 3 Department of Rheumatology, Besançon University Hospital, Besançon, France; 4 Department of Pulmonary Function Testing and Exercise Physiology, University Hospital of Nancy, 54000 Nancy, France; 5 Development, Adaptation and Disadvantage, Cardiorespiratory Regulations and Motor Control (EA 3450 DevAH), University of Lorraine, 54505 Nancy, France; 6 Faculté de Medecine, InSciDens, Université de Lorraine, 54000 Nancy, France; 7 Université de Lorraine, CNRS, IECL, 54000 Nancy, France; 8 CHRU-Nancy, DRCI, Département MPI, Unité de méthodologie, Data management et statistiques UMDS, 54000 Nancy, France; 9 Ingénierie Moléculaire et Physiopathologie Articulaire (IMoPA), UMR 7365 CNRS – University of Lorraine, Nancy, France; 10 Contrat d’Interface, Department of Rheumatology, Nancy University Hospital, Nancy, France

**Keywords:** Medical research, Rheumatology

## Abstract

To evaluate whether the risk of bone fragility on computed tomography (CT) (scanographic bone attenuation coefficient of the first lumbar vertebra (SBAC-L1)) is associated with the severity of spine structural involvement (mSASSS) in patients with ankylosing spondylitis (AS). This retrospective study included AS patients, followed from 2009 to 2017, who fulfilled the New York criteria and who underwent thoraco-abdomino-pelvic CT and radiography (spine, pelvis). The structural involvement was retained for mSASSS ≥ 2. The SBAC-L1 was measured in Hounsfield units (HU). A SBAC-L1 ≤ 145 HU was used to define patients at risk of vertebral fracture (VF). A total of 73 AS patients were included (mean age: 60.3 (± 10.7) years, 8 women (11%), mean disease duration: 24.6 years (± 13.9)). Sixty patients (82.2%) had a mSASSS ≥ 2 (mean score 20.7 (± 21.2)). The mean SBAC-L1 was 141.1 HU (± 45), 138.1 HU (± 44.8) and 154.8 HU (± 44.9) in the total, mSASSS ≥ 2 and mSASSS < 2 populations, respectively. Patients with bone bridges had lower SBAC-L1 than mSASSS ≥ 2 patients without ankylosis (*p* = 0.02) and more often SBAC-L1 ≤ 145 HU (73% vs 41.9%, *p* = 0.006). A SBAC-L1 ≤ 145 HU was not associated with structural spine involvement, but patients with bone bridges had significantly decreased SBAC-L1 and an increased probability of being under the fracture threshold.

## Introduction

Ankylosing spondylitis (AS) is a chronic inflammatory rheumatic disorder with axial (spinal and sacroiliac) and peripheral involvement (arthritis, enthesitis, dactylitis) and sometimes some extra-articular events (iritis, inflammatory bowel disease, dactylitis). In the same patient, bone resorption (erosion, osteoporosis) progressed to bone formation (syndesmophyte, ossification of ligament, ankylosis) and could also be completely disconnected. Then, morbidity and mortality are increased due to vertebral fracture and neurological complications (odds ratio (OR) 3.26^1^).

In AS or spondylarthritis (SpA) patients, the prevalence of osteoporosis screened by DXA ranged from 11.7 to 34% regardless of the location considered^1–9^. The hip or femoral neck T-scores were lower in cases of axial structural involvement and longer disease duration^2,10^. Concerning spine DXA, the results are more controversial: for some authors, the spine T-score was higher for patients with spine structural involvement than for patients without syndesmophytes^2,10^. However, some studies did not find any difference in spine DXA according to spine structural involvement^2,11,12^. Some studies showed that patients with vertebral fracture (VF) had a higher mSASSS than patients without VF^10^, but some studies did not find any association^8^. Due to syndesmophytes or ankylosis, the spine DXA should be performed on a patient on lateral decubitus and not with an antero-posterior incidence^10,13–15^. Vertebral fracture complications are more frequent in this population, with a prevalence between 11 and 24.6% and an OR of 1.98 compared to a control population^9^ and are associated with a higher morbi-mortality.

Thoracic or thoraco-abdomino-pelvic (TAP) computed tomography (CT) allowed an evaluation of the trabecular bone fragility through the scanographic bone attenuation coefficient of the first lumbar vertebra (SBAC-L1)^16^. The SBAC-L1 studied only trabecular bone in contrast to DXA, where bone formation (syndesmophytes, osteophytes…) or calcifications (vascular) may cause artefacts. This coefficient corresponded to the average bone mineral density, in Hounsfield units, of a region of interest (ROI), drawn in the trabecular bone, avoiding cortical bone. A recent study^16^ reported that a SBAC-L1 ≤ 145 HU (Hounsfield units) was more reliable than a T-score ≤ -2.5 SD for identifying a VF. Indeed, the SBAC-L1 identified 96.6% of the patients with VF, whereas the DXA (with a T-score ≤ -2.5 standard deviation (SD)) identified only 39% in a general population. Recently, the diagnostic performance of SBAC-L1 was studied in rheumatoid arthritis. In this population at risk of osteoporosis, 74% of patients with osteoporotic fractures were categorized as osteoporotic with a SBAC- L1 ≤ 135 HU, whereas only 42% were identified by DXA^17^. Furthermore, SBAC-L1 allowed the identification of 50% of sclerodermic patients with spinal bone fragility and was associated with some criteria of disease severity^18^.

CT scans are often performed in patients with AS to evaluate complications or intercurrent events (infectious, cancerous, etc.) associated or not with the consequences of immunosuppressive drugs. The SBAC-L1 measure seems to be an optimized method to study bone fragility of the trabecular bone in AS patients with spinal structural involvement, without the artefacts due to cortical bone or vascular calcifications, as with DXA^10,13–15,19–22^.

Our primary objective was to determine the relationship between SBAC-L1 and spinal structural involvement, as measured by the mSASSS (modified Stoke Ankylosing Spondylitis Spinal Score) in AS patients. Secondly, we studied the risk factors associated with a SBAC-L1 ≤ 145 HU (fracture threshold) and vertebral fracture on spine radiographs.

## Patients and methods

### Population

This work is a descriptive retrospective study performed on patients examined at our Hospital for AS, fulfilling the ASAS 2009 or New York modified criteria, with radiographic sacroiliitis (≥ Grade 2 bilateral or ≥ 3 unilateral) and/or severe sacroiliitis on sacroiliac CT scan, according to the Bennett criteria^23,24^. They must have undergone radiography of the pelvis, cervico-dorso-lumbar spine and a CT scan (thoracic, thoraco-abdomino-pelvic or spinal) including the first lumbar vertebra (L1), with a time between imaging sessions that did not exceed 2 years^25^. The records were selected among spondyloarthritis patients with a screening performed between 2009 and March 2017.

Demographic characteristics (age, gender, smoking…), clinical data (disease duration, activity score…), biological data (C-reactive protein (CRP)) and information about therapeutic treatments (calcium, vitamin D…) were collected from the complete medical records on about AS and osteoporosis^26^. Clinical risk factors of osteoporosis (gender, age, biological inflammation, smoking and corticosteroids) were also collected. Osteoporosis is classically defined as a T-score ≤ -2.5 SD on DXA (standard deviation). We selected DXA and CT scan a maximum period of 2 years apart^26^.

### AS radiographic assessment

The radiographs were anonymised and read in OsiriX software (v6.5.1-64 bits).

The frontal pelvic radiographs or radiographs from the lumbo-sacral junction, are read by 2 independent senior readers, with an adjudication by a third rheumatologist in case of discordance for the diagnosis of structural sacroiliitis, according to the Bennett criteria^23,24^.

The mSASSS score ranged from 0 to 72^27,28^ was performed on sagittal spinal radiographs (cervical and lumbar). The presence of squaring, syndesmophytes and ankylosis (bone bridge) from the inferior corner of the C2 vertebra to the superior corner of the T1 vertebra and from the inferior corner of the T12 vertebra to the superior corner of the S1 vertebra was scored according to the following manner: each anterior corner was graded according to the following manner: grade 1 for squaring; grade 2 for a syndesmophyte, and grade 3 for ankylosis. A structural damage (mSASSS +) was defined by a mSASSS ≥ 2, with the presence of at least one syndesmophyte. We also studied the number and location of bone bridge. The mSASSS was performed by three rheumatologists. The diagnosis of syndesmophyte was considered if 2 of the 3 readers agreed. The mSASSS was calculated after the adjudication for each corner, without considering grade 1, in view of the poor reproducibility of this lesion demonstrated in previous studies^29–31^.

### Vertebral fracture evaluation on radiography

Vertebral fractures were graded and localized on sagittal radiographs of the thoracic and lumbar spine according to Genant’s classification^32^. This study was performed by 3 rheumatologists. The diagnosis of VF was retained if 2 of the 3 lectors agreed for the presence of VF. Intra-reader reproducibility was performed on the whole population.

### Reproducibility of the measures (mSASSS, VF and sacroiliitis)

Intra-reader reproducibility was tested on 30 exams and inter-reader reproducibility on all exams for 2 readers with an additional reader for the adjudication in case of discordance between the 2 initial readers.

### Scanographic bone attenuation coefficient of L1: SBAC-L1 (Fig. 1)

**Figure 1:**
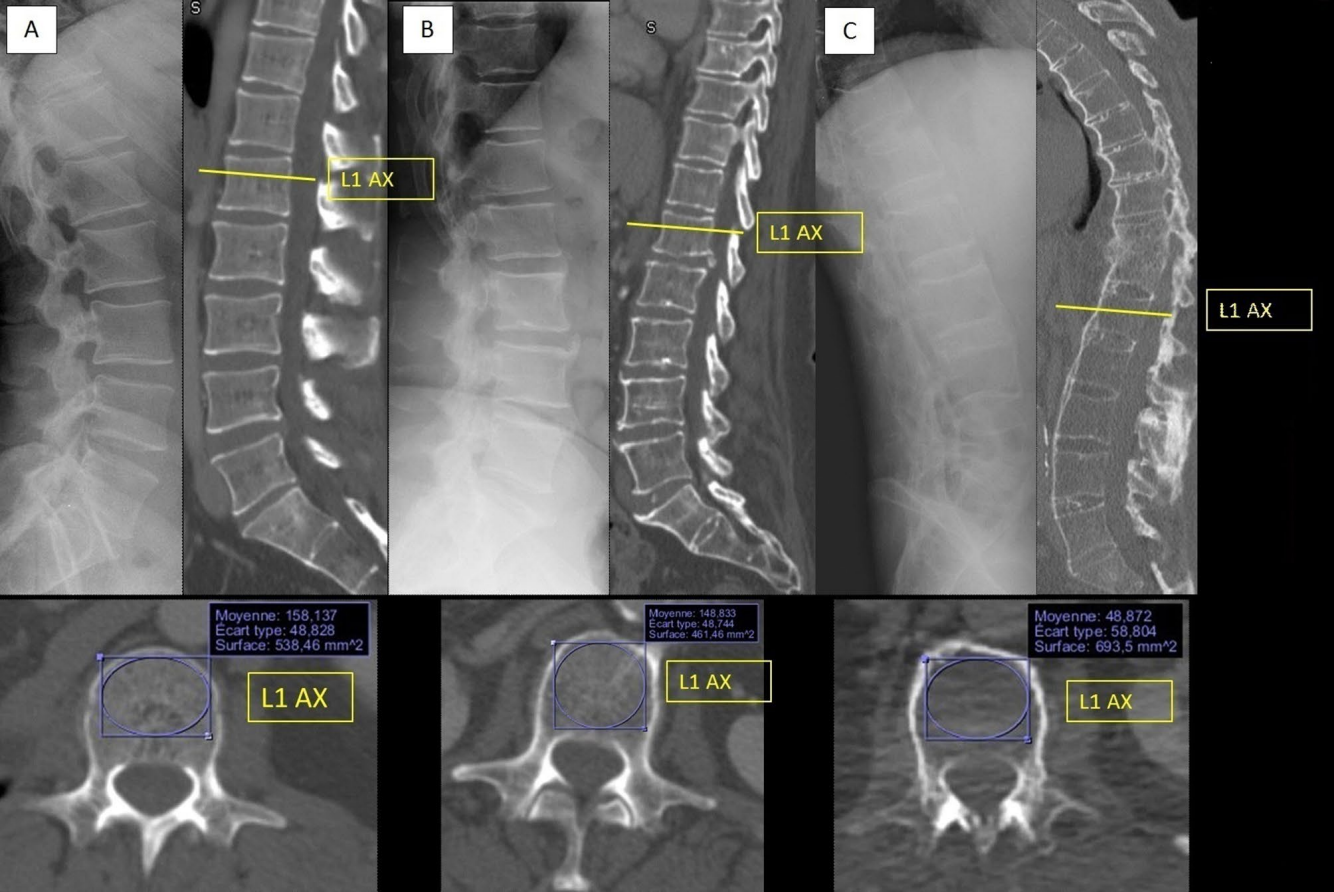
SBAC-L1 measure on normal spine (A), spine with syndesmophytes (B) and ankylosed spine (C). (OsiriX software (v6.5.1-64 bits). https://www.osirix-viewer.com).

All the CT scans were performed at the University Hospital of Nancy and were read using OsiriX software (v6.5.1-64 bits). Most of the CT were performed before a biotherapy. For the other patients, CT were performed for pulmonary symptoms (cough, dyspnea), for deterioration of general condition, for biological inflammation, for a research of neoplasia after fracture… The tube voltage was 120 kVp (if the information was available). We used the axial sections with the available smallest slice thickness, without reconstruction. Multiple row detector array scans were using. The field of exploration included the thoraco-abdomino-pelvic or only abdomino-pelvic regions. The acquisition diameter was 50 cm with a 512 × 512 matrix. Slices varying between 0.6 and 5 mm thick in the axial acquisition mode, with a mean thick of 1.1 mm. These examinations were performed with injection of contrast material unless contra-indicated.

The SBAC-L1 study was conducted on L1 axial sections through the pedicles on the bone window, as previously described^16^. The largest elliptical region of interest (ROI) was drawn in the trabecular bone and provided the mean bone mineral density (in HU). If there was a VF in the L1 vertebra, the measurement was performed on the adjacent vertebrae (CT scan performed similarly from T12 to L5^29^). This evaluation was performed by a single reader (MF) because the excellent reliability of this measure has been previously demonstrated (intra- and inter-reader, kappa > 0.9)^18^.

A threshold of 145 HU was used because it identified 96.6% of the patients with VF in the general population, whereas the DXA (with T-score ≤ -2.5 SD) identified only 39% of VF in the same population^16^. This threshold of 145 HU was used to maintain an acceptable balance between sensitivity and specificity in this population, in which a higher risk of bone fragility is suspected.

### Statistical analysis

Both descriptive and comparative analyses were conducted by accounting for the nature and distribution of the variables. Qualitative variables were described with frequencies and the percentage; quantitative variables were evaluated with the mean ± SD (standard deviation) or with the median and interquartile range (IQR). For quantitative variables, the Student’s t test or the Mann–Whitney U test were used. For qualitative variables, the chi-squared test was used, along with Fisher’s exact test if necessary. Pearson’s coefficient allowed for the analysis of the correlations. Univariate and multivariate analyses are presented with the odds ratio (OR) and its 95% confidence interval (CI 95%).

To analyse the intra-reader and inter-reader reliability, we used Cohen’s kappa method. The risk α was established as 0.05. IBM SPSS Statistics v23 was the software used for the data analysis.

### Ethics approval

All of the data used were obtained from the medical records. No examinations were performed for patients to meet the inclusion criteria. This study is registered to “Ethics committee of the Nancy University Hospital (file number: 2019PI007, chaired by Pr Martinet)” and was designed in accordance with the general ethical principles outlined in the Declaration of Helsinki. The study protocol was approved by “Ethics committee of the Nancy University Hospital chaired by Pr Martinet”. All patients gave their written informed consent for the use of their medical data during the time period they received medical care at the University Hospital, through the signing of the hospital patient charter.

## Results

### Population

Among 1503 spondyloarthritis patients followed between 2008 and 2017 and screened for biologic treatment, 73 patients fulfilled AS inclusion criteria, with a mean age of 60.3 years (± 10.7) and a large predominance of men (89%). The patients presented a mean disease duration of 24.6 years (± 24) and suffered from active disease with disability (Table1). Patients were mainly treated by TNF inhibitors in 58.9%, by NSAIDs in 67.1%, and only 9.6% by corticosteroids (Table 1).

**Table 1:** Demographical, clinical characteristics and bone assessment on CT of the 73 AS patients according to spine structural involvement (mSASSS or vertebra ankylosis).

	All patients n = 73	mSASSS + n = 60	mSASSS - n = 13	*p*	mSASSS +		
					Bone bridge +	Bone bridge -	*p*
					n = 37	n = 23	
**Demographic and clinic**							
Age (n = 73)	60.3 (60)	62 (61)	52.7 (57)	0.493	62 (57.5–73)	59 (53–67)	0.88
Men (n = 73)	65 (89)	53 (88.3)	12 (92.3)	0.677	34 (91.9)	19 (82.6)	0.276
Smoker (n = 48)	31 (42.5)	25 (41.7)	6 (46.2)	0.5	17 (45.9)	8 (34.8)	0.931
Alcool (n = 28)	4 (5.48)	4 (6.7)	0 (0)	0.678	4 (10.8)	0 (0)	0.160
**AS characteristics**							
Disease duration (n = 71)	24 (12–34)	25 (13–35)	15.5 (12–25.25)	0.076	27 (13.75–35.75)	24 (11–35)	0.529
BASFI (n = 58)	43.5 (23.7)	44.5 (22.2)	41.2 (30.8)	0.598	46.7 (22.2)	42 (22.4)	0.319
BASDAI (n = 62)	7.2 (10.5)	7.1 (10.5)	8.4 (11.3)	1.0	7.2 (12.7)	6.9 (7.3)	0.236
ASDAS (n = 21)	3.4 (1.2)	3.4 (0.9)	3 (2.6)	0.763	3.7 (1)	2.81 (0.6)	**0.035**
HLA B27 (n = 69)	46 (63)	39 (65)	7 (53.8)	0.276	26 (70.3)	13 (56.5)	0.075
Biological inflammation CRP (n = 69)	31 (42.5)	28 (46.7)	3 (23.1)	0.079	18 (48.6)	10 (43.5)	0.415
mSASSS on radiographs (n = 73)	20.7 (21.2)	25.1 (20.8)	0 (0)	**0.0001**	37.2 (17.8)	5.9 (4.2)	**0.0001**
**Treatments**							
Corticosteroids (n = 49)	7 (9.6)	5 (8.3)	2 (15.4)	0.243	3 (8.1)	2 (8.7)	0.926
NSAIDs (n = 51)	49 (67.1)	44 (73.3)	5 (38.5)	0.634	30 (81.1)	14 (60.9)	**0.048**
Proton Pomp Inhibitor (n = 15)	15 (20.5)	14 (23.3)	1 (7.7)	/	9 (24.3)	5 (21.7)	/
TNF inhibitor (n = 43)	43 (58.9)	36 (60)	7 (53.8)	/	23 (62.2)	13 (56.5)	/
Vitamin D and/or calcium (n = 73)	18 (24.6)	14 (23.3)	4 (30.8)	0.573	9 (24.3)	5 (21.7)	0.818
Specific treatment for osteoporosis (n = 72)	12 (16.4)	9 (15)	3 (23.1)	0.493	8 (21.6)	1 (4.3)	0.063
**SBAC-L1**							
Mean (SD) in HU	141.1 (45)	138.1 (44.8)	154.8 (44.9)	0.239	123.96 (41.1)	160.4 (41.9)	**0.002**
Fracture threshold (145 HU)	42 (57.5)	36 (60)	6 (46.2)	0.325	27 (73)	9 (39.1)	**0.006**
**VF**							
Number of patients	9 (12.3)	8 (13.3)	1 (7.7)	0.575	5 (13.5)	3 (13)	0.958
Number of fractures	13	12	1	0.582	8	4	0.435

all data in bold are statiscally significant data (p<0.05)

AS, ankylosing spondylitis; ASDAS, Ankylosing Spondylitis Disease Activity Score; BASDAI, Bath ankylosing spondylitis disease activity index; BASFI, Bath ankylosing spondylitis functional index; CRP, C-reactive protein; HU, Hounsfield unit; mSASSS, modified Stoke Ankylosing Spondylitis Spinal Score; NSAIDs, Non-steroidal anti-inflammatory drugs; SBAC-L1, scanographic bone attenuation coefficient of the first lumbar vertebra; SD, standard deviation; TNF, tumour necrosis factor; VF, vertebral fracture.

Age in mSASSS + group and disease duration are in median (IQR), whereas age in general population, BASDAI, BASFI, ASDAS are in mean (± SD). In the comparisons, the Student’s t-test or the Mann–Whitney U test were used. For qualitative variables, the chi-square test with, if necessary, the exact calculation of Fisher was used. The risk α was established as 0.05.

mSASSS + was defined by a mSASSS ≥ 2, with the presence of at least one syndesmophyte.

The mean duration between radiographs and CT scans was 75.6 days. For DXA and CT scan, the mean duration between these two exams was 128.2 days.

Concerning the risk of osteoporosis, 53 patients (72.6%) have at least one clinical or biological risk factor for osteoporosis. In the total population, 11 (15.07%) take calcium, 16 (21.9%) take vitamin D and 12 (16.4%) were on a specific treatment for osteoporosis (Table 1).

### AS radiographic assessment

**Figure 2:**
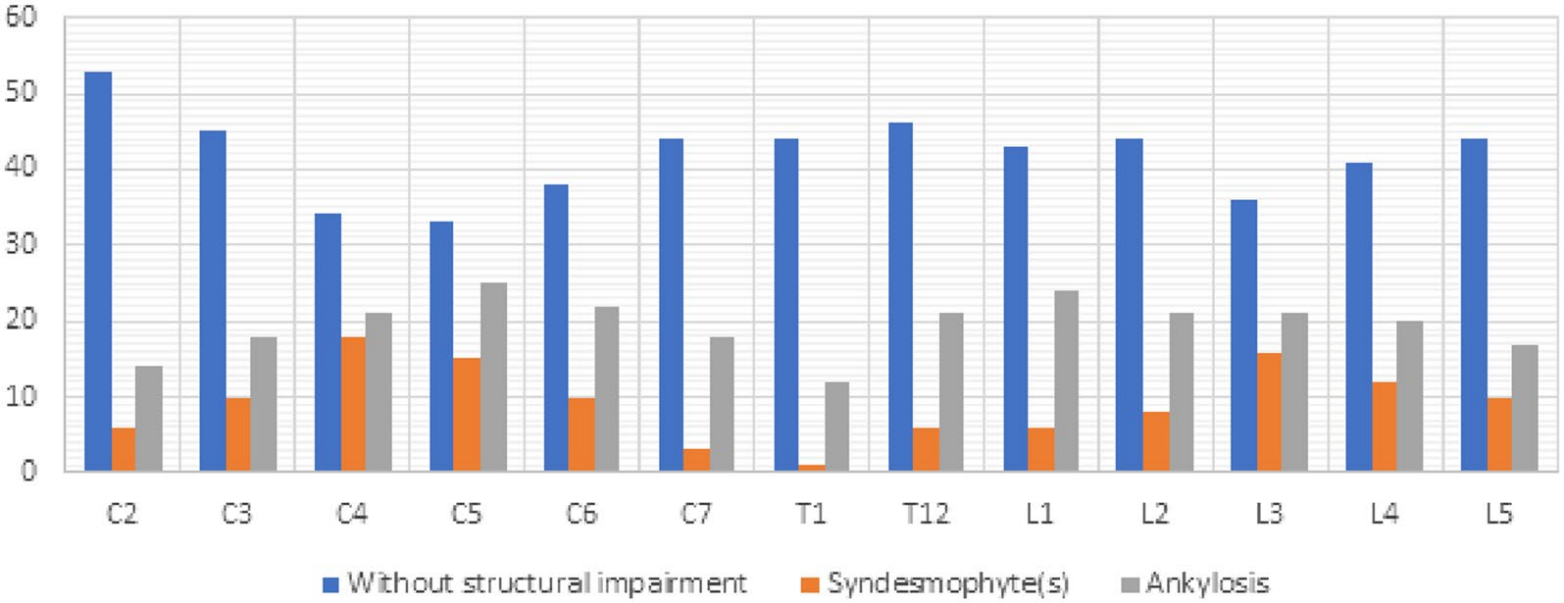
Spine structural lesions for each vertebra issued from mSASSS analysis.

All patients presented sacroiliitis on radiography, and 60 of them (82.2%) had a mSASSS ≥ 2 (at least one syndesmophyte). The cervical spine assessment was technically limited in patients with cervical spine ankylosed in kyphosis position. Thus, an optimal radiographic assessment of the cervical spine was available for 79.5% of the AS patients. For lumbar assessment, all vertebrae were available. The mean mSASSS was 20.7 (± 21.2), 25.1 (± 20.8) and 37.2 (± 17.8) in the total AS population, in AS patients with mSASSS + and in AS patients with bone bridges, respectively (Table 1). Finally, 37 AS patients (50.7%) presented at least one bone bridge on the spine (Fig. 2). C3 and L3 vertebrae were the most prevalent ankylosed vertebrae.

### Vertebral fracture evaluation on radiography

On radiography, 13 vertebral fractures were observed in 9 patients (12.3%): 7 patients with one FV, 1 patient with 2 FV and one patient with 4 FV. Only one patient presented a L1 VF. For this patient, the SBAC was studied on L2. 8 patients were mSASSS + , one mSASSS – (*p* = 0.575) (Table 1); seven VF are Grade 1, five Grade 2 and only one Grade 3 according to Genant’s classification and were localized on thoracic or lumbar vertebrae.

### Reproducibility of the measures (mSASSS, VF and sacroiliitis)

The reproducibility, intra- and inter-reader, for the structural involvement (on spine and on sacroiliac joints) was good to excellent. For mSASSS, the kappa coefficient was 0.97 (IC95%: 0.93–0.98) and 0.97 (IC95%: 0.95–0.98), for intra- and inter-reader respectively. For sacroiliac joint, the kappa coefficient was 0.71 (IC95%: 0.49–0.89) and 0.82 (IC95%: 0.7–0.93) for the right and 0.54 (IC95%: 0.30–0.75) and 0.69 (IC 95%: 0.55–0.82) for the left. For VF, the reproducibility was moderate to fair; the kappa coefficient was 0.5 (IC95%: 0.14–0.82) and 0.29 (IC95%: 0.04–0.55) for intra- and inter-reader respectively.

For VF, an adjudication was needed for 15 patients: 4 were without VF and 11 with VF after adjudication.

For the right sacroiliitis (SI), we find 7 patients needed an adjudication. For 3 of them, the adjudication tended to the lower grade of sacroiliitis. For the left SI, 15 needed adjudication and 9 adjudications tended to the lower grade of sacroiliitis.

For mSASSS, the adjudication permitted to reclass 3 patients in mSASSS- group.

### Scanographic bone attenuation coefficient of L1: SBAC-L1

Sixty-seven patients had thoraco-abdomino-pelvic CT scans, 3 patients had thoracic CT scans and 3 patients had abdomino-pelvic CT scans. Only 7 patients did not received contrast.

**Figure 3:**
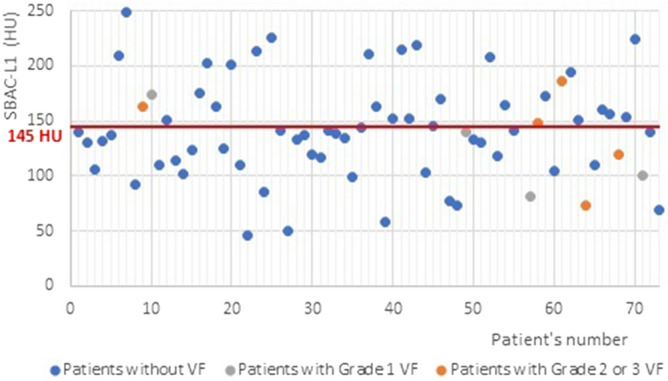
SBAC-L1 according the presence of VF.

In the whole population, the mean SBAC-L1 was 141.1 HU (± 45). In mSASSS + , the mean SBAC-L1 was 138.1 HU (± 44.8) and 154.8 HU (± 44.9) in mSASSS—(*p* = 0.24). Forty-two patients (57.5%) had a SBAC-L1 ≤ to the fracture threshold (145 HU), 36 (60%) had a mSASSS + and 6 (46.2%) had a mSASSS—(*p* = 0.325). Among patients with bone bridges, 27 (73%) had a SBAC-L1 ≤ 145 HU and only 39.1% without bone bridges (*p* = 0.006). The presence of at least one bone bridge was associated with a significant decrease in SBAC-L1 calculated at 123.9 HU (± 41.1) (*p* = 0.002). For the 9 patients with VF, 5 (55.6%) had SBAC-L1 under the fracture threshold (Fig. 3).

### Risk factor of osteoporosis associated with mSASSS + 

No clinical or demographical characteristics were associated with SBAC-L1 ≤ 145 UH, VF or mSASSS + . Only elevated ASDAS was associated with mSASSS + in the univariate analysis (*p* = 0.035).

### Correlation between mSASSS and SBAC-L1

There was no correlation between mSASSS and SBAC-L1 in the total population and in sub-groups (mSASSS + /mSASSS-) (R^2^ 0.21 for general population and 0.24 for mSASSS +).

### Correlation between SBAC-L1 and the number of bone bridges

There was no correlation between SBAC-L1 and the number of bone bridges (R^2^ = 0.13).

## Discussion

The prevalence of VF was 12.3% in AS patients, and VF was mainly observed in mSASSS + patients (88.9%). A SBAC-L1 under the fracture threshold was not associated with structural spine involvement (evaluated by mSASSS), but patients with bone bridges had significantly lower SBAC-L1 and more of them were under the fracture threshold.

This study was performed in an AS population with similar demographic and clinical characteristics, such as GESPIC and OASIS cohorts^33,34^. In the GESPIC cohort, the population (n = 120) was composed of a mixed of SpA and AS patients, while in the OASIS cohort, 210 AS patients with sacroiliitis were followed. In our population, patients were more often treated with TNF inhibitor (58.9%), but the prevalence of NSAIDs was approximately 67%. We know that TNF inhibitors may play a protective effect on bone^35^. In our population, 58.9% of the patients were treated with TNF-blockers without a difference between mSASSS− and mSASSS+ . Concerning structural spinal involvement, 18.7% of our patients did not have spinal structural lesions (mSASSS-). This prevalence is in agreement with the OASIS cohort, in which 19% of the patients were mSASSS−^33^. Our mean mSASSS score was calculated at 20.7 versus 10.8 in the OASIS cohort. Higher mSASSS in our population is probably due to a longer disease duration in older patients with a predominance of males^33^. Low value of SBAC-L1 in these old patients could also be influenced by a long disease duration. In these two cohorts, the osteoporosis comorbidity and their treatments were not evaluated. The rate of AS patients being positive for HLA B27 (63%) is rather low, but we have no specific explanation for this low rate.

In our population, we found at least one clinical risk factor for osteoporosis (menopausal women, corticotherapy, alcohol or smoking, biological inflammation) in 72.6% of the patients. The prevalence of VF was 12.3%. This prevalence is concordant with the literature data^9,19,36^, except with the DESIR cohort, which showed a lower prevalence than other studies (only 3%), probably due to younger patients (34.3 ± 8.7 years), with shorter disease duration (1.5 ± 0.9 years) and less structural sacroiliitis (13.5%) or spinal involvement (7%)^36–38^.

In clinical practice, osteoporosis is probably underestimated, since only one-quarter of our patients were screened by DXA. Klingberg^8^ demonstrated that spine was the most common location of osteoporosis in AS patients with low BMD correlated with higher mSASSS scores. However, the lumbar BMD performed on antero-posterior projection was significantly higher than DXA performed on lateral projection (*p* < 0.001). Similar results were also reported by Karberg et al.^39^. However, most studies^12,13,15,21,40^ mentioned more osteoporosis at the hip and femoral neck in AS patients with spine involvement. In these patients, BMD of the spine measured by DXA was higher than in patients without spine lesions. These DXA was performed with antero-posterior and lateral projections for 2 studies^12,15^, only antero-posterior projections for one study^21^ and for 2 studies, the spine DXA evaluation method was not explained^13,40^. For these reasons, osteoporosis screening by DXA is more pertinent for the hip and is still debated in the spine, even if the lateral projection of the lumbar spine is most likely more pertinent ^10,13–15,21,22,30,36^, but this measurement is not recommended, due to huge problems of reproducibility, and the lack of reference values.

On CT, the SBAC-L1 measure was limited to the trabecular bone avoiding cortical proliferation, such as syndesmophytes and bone bridge formation, as well as extra-vertebra calcifications. We showed that 57.5% of the patients were under the threshold of bone fragility. mSASSS + patients tended to have a lower SBAC-L1 compared to mSASSS-, but the difference was not significant due to a lack of power. The lack of association resulted from the small number of mSASSS- patients. Moreover, we showed for the first time that SBAC-L1 was lower in the case of a bone bridge (*p* = 0.002) compared to patients with mSASSS + without a bone bridge. The main pathophysiology hypothesis is a lower strain on vertebrae and as a consequence on bone when there is ankylosis. Moreover, bone ankylosis may also reflect patients with more severe disease with less physical activity due to pain or loss of mobility increasing the bone loss^41–45^. Further studies should be performed on a larger sample to elucidate the impact of each of these parameters.

Modifications of the trabecular bone in AS patients have also been reported by high-resolution peripheral quantitative computerized tomography (HRpQCT) in the AS population compared to controls^22^. Authors reported significantly lower volumetric bone mineral density (vBMD) in cortical bone of the ultradistal radius (*P* = 0.007) but also in trabecular bone of the ultradistal tibia (*P* = 0.033). Quantitative computed tomography (QCT) of the lumbar vertebra also demonstrated lower volumetric trabecular bone mineral density trabecular, indicating poor bone microarchitecture, with thinner trabeculae, lower trabecular number, lower cortical vBMD, and increased cortical porosity. These results suggested that trabecular bone fragility may also be related to a general process since in these cases, no ankylosis was reported^22^. The SBAC-L1 evaluation is a modern method to estimate the fracture risk but it is less precise than original QCT values that use a special phantom for the calibration. In AS patients, low trabecular bone score (TBS) could predict major osteoporotic fractures independent of FRAX^46,47^. TBS was negatively correlated to mSASSS in AS patients^48^.

The reproducibility values of our measures were very good for mSASSS and for SBAC-L1 measurements^26^. The results are similar in the literature on radiography as well on CT, which supports the possibility of using SBAC-L1 in clinical practice^18,49^. Concerning the use of contrast, Pickhardt found negligible differences in mean L1 attenuation values according to the administration of intravenous contrast agent^16,50^. However, the reproducibility of VF evaluation was moderate to poor according to the lectors (2 moderate inter-readers, one poor inter-readers and one moderate intra-readers reproducibility). In the literature, the reproducibility of the VF study with Genant’s classification is slightly better^32,51^, but the moderate reliability is probably due to the difficulty in distinguishing cuneiform vertebrae from vertebral fracture of grade 1 in spine with or without kyphosis.

Concerning VF, regarding the small number of patients, the small number of VF, the poor reliability in the identification of VF on radiographs and the complex pathogenesis of fracture in AS patients as compared to the general population, some studies with higher patients number are needed to study the pathophysiological mechanisms.

## Conclusion

In conclusion, the prevalence of VF was 12.3% in AS patients, and VF was mainly observed in mSASSS + patients (88.9%). A SBAC-L1 under the fracture threshold was not associated with structural spine involvement (assessed by mSASSS), but patients with bone bridge had significantly lower SBAC-L1 and more of them were under the fracture threshold.

## Data Availability

The datasets generated during and/or analyzed during the current study are available from the corresponding author on reasonable request.
